# *Ralstonia solanacearum* Facing Spread-Determining Climatic Temperatures, Sustained Starvation, and Naturally Induced Resuscitation of Viable but Non-Culturable Cells in Environmental Water

**DOI:** 10.3390/microorganisms10122503

**Published:** 2022-12-16

**Authors:** Belén Álvarez, María M. López, Elena G. Biosca

**Affiliations:** 1 Departamento de Bacteriología, Instituto Valenciano de Investigaciones Agrarias (IVIA), 46113 Valencia, Spain; 2 Departamento de Microbiología y Ecología, Universitat de València (UV), 46100 Valencia, Spain

**Keywords:** bacterial wilt, global warming, environmental stress, VBNC, pathogenicity

## Abstract

*Ralstonia solanacearum* is a bacterial phytopathogen affecting staple crops, originally from tropical and subtropical areas, whose ability to survive in temperate environments is of concern under global warming. In this study, two *R. solanacearum* strains from either cold or warm habitats were stressed by simultaneous exposure to natural oligotrophy at low (4 °C), temperate (14 °C), or warm (24 °C) temperatures in environmental water. At 4 °C, the effect of temperature was higher than that of oligotrophy, since *R. solanacearum* went into a viable but non-culturable (VBNC) state, which proved to be dependent on water nutrient contents. Resuscitation was demonstrated *in vitro* and *in planta*. At 14 °C and 24 °C, the effect of oligotrophy was higher than that of temperature on *R. solanacearum* populations, displaying starvation-survival responses and morphological changes which were stronger at 24 °C. In tomato plants, starved, cold-induced VBNC, and/or resuscitated cells maintained virulence. The strains behaved similarly regardless of their cold or warm areas of origin. This work firstly describes the natural nutrient availability of environmental water favoring *R. solanacearum* survival, adaptations, and resuscitation in conditions that can be found in natural settings. These findings will contribute to anticipate the ability of *R. solanacearum* to spread, establish, and induce disease in new geographical and climatic areas.

## 1. Introduction

The plant pathogen *Ralstonia solanacearum* is a relevant species and former constituent of the *R. solanacearum* species complex [1,2,3]. It causes severe wilt disease and economic losses in solanaceous and other basic crops for human consumption worldwide as well as in important ornamentals [4,5,6,7,8]. The pathogen frequently cannot be effectively controlled due to its high pathogenic potential and persistence in natural settings. Bacterial wilt control in the field has frequently been addressed by conventional methods, mainly agrochemicals and/or cultural practices, with variable results, and often with environmental impact [9,10]. Alternatively, biological control methods are being explored, such as bacteriophage-based treatments. The use of lytic bacteriophages may be an eco-sustainable strategy because of their specificity and bactericidal activity, although until now no bacteriophage-based product is commercially available against *R. solanacearum* [10]. Therefore, this pathogen poses a threat to the maintenance of global food security. In fact, the species has a quarantine status in the European Union (EU), the USA and Canada [5,11,12], and is considered a priority pathogen in agriculture for control and containment [13]. A major concern is that *R. solanacearum* seems to hold great potential for geographical expansion even under environmentally unsuitable conditions, as it appears to infect plants and persist during variable periods in soil or surface water as a free-living form or associated to plant material or non-host roots [4,14,15,16,17]. This is despite the exposure to abiotic stresses compromising the endurance of the bacterium, such as sustained oligotrophy and sub-optimal temperatures. In water systems, it can be consistently detected for years after its introduction, maintaining pathogenicity [16,17,18,19,20,21,22]. This creates a problem for farmers, as water is a scarce resource, particularly under the current conditions of global warming. According to EU and other countries’ regulations, there is a ban on the irrigation of host plants with *R. solanacearum*-contaminated water as long as the bacterium is detected. The procedure for detection is mainly based on the molecular identification of *R. solanacearum* colonies isolated from the water. Global climatic changes are thought to increase extreme climate events and the prevalence of abiotic stresses. It is therefore decisive to understand the impact of prevailing environmental factors on *R. solanacearum* persistence in watercourses to be able to foresee changes regarding water-borne dissemination of the pathogen [6,8,12] and colonization of new geographical and climatic areas.

In that sense, knowledge about the effects of environmental temperatures on microorganisms is crucial to understand bacterial growth and adaptations facing global warming, as well as pathogen virulence and expression of symptoms in the plant [23]. Further, increased temperature is frequently associated with increased severity of the bacterial wilt disease [14,24,25]. Likewise, in watercourses, *R. solanacearum* population levels seasonally varied according to a range of temperatures [15,16,17], and persistence was also variable in agricultural water microcosms [26]. *R. solanacearum*, considered a pathogen of tropical and subtropical regions, is capable of causing bacterial wilt in temperate latitudes [27]. However, results from epidemiological studies are contradictory regarding the ability of *R. solanacearum* to survive in cold conditions away from plants [28]. Although the pathogen becomes viable but non-culturable (VBNC) in pure water by prolonged exposure to 4 °C [26,28,29], the fact that up to date the dynamics of this process has not been monitored in more realistic environmental water microcosms limits the extent to which the results can be extrapolated to the field [28].

Not only temperature, but also the limited nutrient availability characteristic of environmental water has long been claimed to affect bacterial survival in natural settings [30,31]. Weather events are likely to increase the impact of nutrient availability on bacterial communities in the environment. Without climate adaptation strategies, bacteria will probably have to either disseminate or stay and face starvation. *R. solanacearum* can persist in water for different periods [26,29,32,33,34,35], remaining pathogenic up to four years in environmental water at a favorable temperature through strategies, such as starvation-survival responses, the VBNC state, transition to coccoid cells, and aggregation [6], mechanisms evolved by non-sporulating bacteria facing adverse environmental conditions [30,31,36,37]. However, the effects of the simultaneous exposure to environmental abiotic stresses, such as different climatic temperatures and starvation on this pathogen survival and adaptation capability in natural environmental water have not yet been clarified.

Furthermore, after induction to the VBNC state by exposure to low temperature, transition to a fully culturable and pathogenic state or resuscitation may occur in favorable environmental conditions [37,38], although evidence for this requires a clear-cut distinction between true resuscitation (reversion from non-culturability to culturability) and regrowth (multiplication of a few culturable cells that had remained undetected). In fact, attempts to resuscitate cold-induced VBNC *R. solanacearum* in water have been carried out by the addition of hydrogen peroxide-degrading compounds, such as catalase to standard solid media to release the pathogen from oxidative stress [28,29]. However, *R. solanacearum* resuscitation by a simple reversal of low temperature has not been documented so far, despite being the main environmental VBNC inducing factor in natural settings, and a key factor in bacterial wilt outbreaks. This finding could have relevant epidemiological consequences under global warming, since increases in water temperature could lead to increased geographical expansion and/or incidence of water-borne infections resulting from resuscitated cells of this phytopathogenic bacterial species.

This work addressed, for the first time, the simultaneous effect of different temperatures and starvation on stress induction of two *R. solanacearum* strains (former *R. solanacearum* phylotype II) from either cold or warm habitats, and their responses in environmental water microcosms. Further, the capability of these *R. solanacearum* strains to resuscitate and keep pathogenic under conditions that can be found in natural settings was also firstly demonstrated. Knowledge derived from this work will help to foresee tendencies in *R. solanacearum* persistence and dissemination in aquatic systems within the frame of global warming, as well as their capability for establishment and disease induction in new geographical and climatic areas.

## 2. Materials and Methods

### 2.1. Bacterial Strains and Culture Conditions

Two bacterial strains of the present species *R. solanacearum* [2,3] isolated from either warm or cold habitats were used: strain IVIA-1602.1, from a diseased potato tuber from Canary Islands (Spain), and strain IPO-1609, from a diseased potato plant from The Netherlands [29], both are race 3, biovar 2 of the former *R. solanacearum* phylotype II. They were kept at −80 °C in a 30% (*v/v*) glycerol medium and routinely grown on the non-selective Yeast Peptone Glucose Agar (YPGA) [39] for 72 h at 29 °C. In stress induction assays, bacterial culturability was tested on YPGA and the Semiselective Medium South Africa (SMSA) agar developed for *R. solanacearum* isolation [40] after incubation at 29 °C for 72 h. SMSA medium was also used to re-isolate the pathogen from the host (tomato plants). Both media are frequently used for *R. solanacearum* isolation, since the colonies of the pathogen can be easily recognized as typically smooth. YPGA contains filtered-sterilized glucose, from which *R. solanacearum* produces a large amount of extracellular polysaccharide. Colonies are fluidal with pearly cream-white whorls. With respect to SMSA, semiselectivity is mainly based on the action of four antibiotics (penicillin, polymyxin, chloramphenicol and bacitracin), triphenyl-tetrazolium chloride, and crystal violet. Colonies are fluidal with reddish whorls.

### 2.2. Characteristics of Environmental and Distilled Water Samples

River water samples were collected according to [16] from four different locations in Spain, and nutrient contents were separately determined for each of them. In the different water samples, organic matter levels were from 2 to 3.73% (*w/v*), and the main ion concentrations ranged as follows (values per liter): Na^+^, 9.7–9.9 mg; K^+^, 2.1–2.9 mg; Ca^2+^, 10.1–13.0 mg; Mg^2+^, 3.9–5.0 mg; dissolved Fe, 0.24–0.27 mg; Mn, 0.06–0.11 mg; Cu, <0.024 mg; dissolved Zn^2+^, <0.018 mg; CO_3_^2−^, <1.8 mg; NO^3−^, 4.37–5.93 mg; P_2_O_5_, 0.374–0.583 mg, and Cl^−^, 9.4–11.4 mg. Salt contents in the samples were correspondent with conductivity values from 151 to 168 µSiemens/cm at 20 °C, and pH values were from 7.48 to 7.83. Distilled water, used for comparative purposes in some assays, had no organic matter and only trace mineral ions: at 20 °C, conductivity was ≤20 µSiemens/cm, and pH value was 7. All water samples were stored in the refrigerator.

### 2.3. Preparation and Monitoring of Stressed R. solanacearum in Water Microcosms

All water samples were autoclaved and filtered through 0.22-µm-pore-size membranes and used for microcosm preparation and inoculation with either of the strains IVIA-1602.1 or IPO-1609 at a range of 5 × 10^6^–1 × 10^7^ CFU (colony-forming units)/mL similarly to [6]. To induce stressed *R. solanacearum* populations, cells in microcosms were incubated at 4 °C, 14 °C, and 24 °C without shaking for 40 days or until loss of culturability. The temperature of 4 °C was selected because it had induced the VBNC state in *R. solanacearum* (former *R. solanacearum* phylotype II) in non-environmental pure water [26,28,29]. The temperatures of 14 °C and 24 °C were within the range in which *R. solanacearum* had been detected in environmental water [16,17]. Initially, microcosms were prepared with each of the four river water samples from different locations, and the survival of *R. solanacearum* monitored at 4 °C and 24 °C. Based on the results obtained, one river water sample was selected for a comparative study on the survival of *R. solanacearum* in microcosms of river water *versus* distilled water at 4 °C, 14 °C, and 24 °C. Microcosms from river water samples and distilled water were prepared in triplicate.

Sampling from each microcosm was performed at inoculation time (day 0) and at 1, 2, 4, 8, 14, 28, and 40 days post-inoculation (dpi) to monitor:

#### 2.3.1. Total, Viable, and Culturable Bacterial Populations

Microscopic counts of total and viable *R. solanacearum* cells were done by a direct viable count (DVC) method [41], extended to 16 h [26] and subsequent staining with either the polyclonal antiserum 1546-H IVIA against *R. solanacearum* or acridine orange [16,42]. Plate counts of culturable cells were done on two media, the general YPGA and the semiselective SMSA, both recommended by EU legislation to isolate the pathogen from environmental samples [43]. *R. solanacearum* colonies for culturable cell counts were confirmed by PCR as described [43] with primers Ps-1 and Ps-2 based on the sequence of the *16S rRNA* gene.

#### 2.3.2. Cell Morphology

Bacterial cell shape was observed by specific immunofluorescence staining with the polyclonal antiserum 1546-H IVIA against *R. solanacearum* [16]. Cell morphology was observed with a Nikon Eclipse E800 microscope at a magnification of ×1000. Pictures were taken with an adapted digital camera DXM1200 using ACT-1 version 2.62 software, and no processing of the images was performed. At each sampling time and for each temperature and environmental water microcosm, the number of bacilli and/or cocci from at least 20 random fields was counted (approximately 300 cells).

#### 2.3.3. Pathogenicity

The ability of starved *R. solanacearum* cells incubated in the environmental water microcosms at 4 °C, 14 °C and 24 °C to induce disease was tested from each triplicate microcosm at each sampling time on groups of 72 tomato plants cv. ‘Roma’ aged three weeks (two plants per microcosm, six plants per water sample at each temperature). Inoculations were performed by injecting into the stem volumes of 10 µL directly taken from the microcosms. Plant inoculations were carried out according to EU Legislation [43]. Positive and negative controls were performed on groups of 12 tomato plants cv. ‘Roma’ (six plants per positive/negative control) at each sampling time. In the case of positive controls, inoculations were performed by injecting 10 µL of a freshly growing cell suspension from either of the two *R. solanacearum* strains. Each suspension was previously washed and adjusted in sterile 10 mM phosphate buffered saline solution (PBS), pH 7.2 (NaCl, 8 g/L; PO_4_H_2_Na·2H_2_O, 0.4 g/L; PO_4_HNa_2_·12H_2_O, 2.7 g/L) to OD_600nm_ = 0.1 (approximately 10^8^ CFU/mL), and diluted to a final concentration of about 10^7^ CFU/mL. In the case of negative controls, inoculations were done by injecting 10 µL of sterile 10 mM PBS. Incubation of the plants and monitoring of disease symptoms were performed in a growth chamber (16 h light, 8 h dark; 26 °C) under quarantine conditions. The pathogen was re-isolated from the wilting plants by cutting 2–3 cm of the stems above the inoculation point, and plating the obtained extracts onto SMSA. The colonies were PCR-identified as described [43] with primers Ps-1 and Ps-2 based on the sequence of the *16S rRNA* gene. Stems from inoculated non-wilted plants were processed in the same way.

### 2.4. Resuscitation of R. solanacearum Populations from the VBNC State Induced in Environmental Water Microcosms

Assays for resuscitation were performed in three different conditions with VBNC *R. solanacearum* populations from the environmental water microcosms at 4 °C in triplicate. To determine if the appearance of culturable cells was due to true resuscitation instead of regrowth of a few remaining culturable but undetected cells, serial ten-fold dilutions were carried out with the VBNC cells, as described [44,45]. The first series of ten-fold dilutions was performed when the microcosms were containing initially approximately 10^6^ viable cells/mL and <10 culturable cells (CFU)/mL, until reaching concentrations of 10^−2^ viable cells/mL and <10^−7^ culturable cells (CFU)/mL. From then and prior to the resuscitation assays, non-culturability was tested at each sampling time by plating volumes of 1 mL directly taken from the microcosms. Resuscitation assays were performed with aliquots (1–10 mL) taken from each environmental water microcosm and their ten-fold dilutions, both *in vitro* and *in planta*, according to three different procedures, as follows:

#### 2.4.1. By Enrichment in a Modified Wilbrink (WB) Broth [16]

Direct aliquots and their ten-fold dilutions in WB broth were incubated at 29 °C with shaking (200 r.p.m.) until appearance of turbidity or for at least one week. Moreover, additional aliquots were taken, transferred to WB broth, and maintained at 4 °C without shaking. Sampling was at time 0 and once a week during a month. To check culturability from turbid tubes, streaks were plated onto YPGA. To test non-culturability from non-turbid tubes, 100-µL volumes were plated onto YPGA. Colonies appeared on plates were PCR-identified. To prove pathogenicity of the colonies on plates, bacterial suspensions were prepared from them and 10-µL volumes were stem-inoculated onto tomato plants (two plants per suspension). Moreover, 10-µL volumes were directly taken from the turbid tubes and inoculated onto stems (two plants per tube) for pathogenicity tests of the cells. Plants were processed for re-isolation and identification of the bacterial pathogen as abovementioned.

#### 2.4.2. By Temperature Upshift in Environmental Water

Briefly, 10-mL aliquots and their ten-fold dilutions in sterile environmental water were incubated at 24 °C without shaking. Sampling was at time zero and each two weeks during a month. To check culturability, 100-µL volumes from the dilutions were daily plated onto YPGA until appearance of colonies or for at least one week. The colonies were PCR-identified and their pathogenicity tested as abovementioned. Moreover, pathogenicity of the cells in the ten-fold dilutions was proved by inoculating 10-µL volumes directly from the dilutions onto the stems (two plants per dilution), which were processed as described.

#### 2.4.3. *In Planta*

From direct aliquots and their ten-fold dilutions in sterile environmental water, 10-µL volumes were stem-inoculated onto tomato plants aged three weeks (two plants per dilution). Sampling was at time 0 and over one month. Plants were processed for re-isolation and identification of the pathogen as abovementioned.

### 2.5. Statistical Analysis

Survival assays were performed at three incubation temperatures with environmental water sample and distilled water in triplicate *R. solanacearum*-inoculated microcosms. Total, viable, and culturable data of *R. solanacearum* cell counts were normalized by log-transformation, and mean values analyzed by a linear regression model considering the following factors: incubation temperature, type of water (environmental or distilled), period of incubation, media, and bacterial strain. Differences among means of coccoid percentages at the three temperatures were estimated by variance analysis (ANOVA). A *p* value <0.05 was defined as significant.

## 3. Results

### 3.1. R. solanacearum Goes into a Nutrient-Dependent Cold-Induced VBNC State in Environmental Water

**Figure 1:**
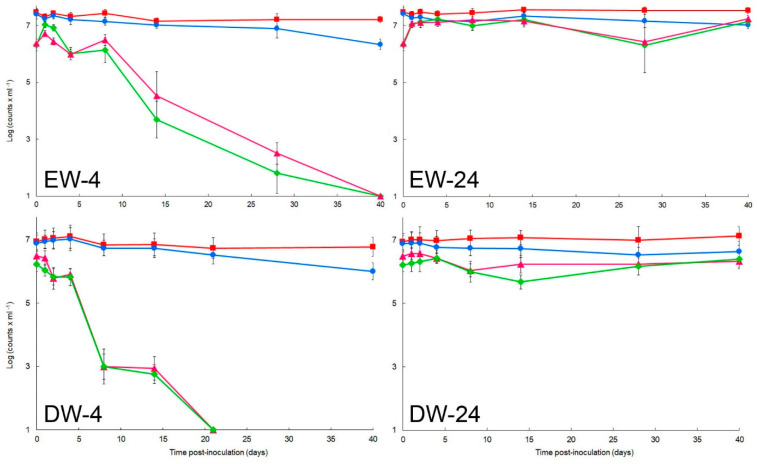
Effect of low temperature under nutrient-limiting conditions on survival of *Ralstonia solanacearum* strain IVIA-1602.1 during 40-day periods in water. Microcosms of: EW−4, environmental water at 4 °C; DW−4, distilled water at 4 °C; EW−24, environmental water at 24 °C, and DW−24, distilled water at 24 °C. Total (■), viable (●), and culturable cells on SMSA (▲) and YPGA (♦) media. Data from one representative environmental water sample have been plotted. Points are mean ± standard deviation of triplicate microcosms.

At low (4 °C) temperature, in environmental or distilled water (Figure 1), total populations of the strain IVIA-1602.1 of *R. solanacearum* remained above their initial inoculation numbers throughout the 40-day experiments, while viability was slightly lower, with declines approximately from 25–30 dpi in both types of water. In contrast, culturable bacterial populations significantly decreased (*p* < 0.05) about one log unit up until eight and four dpi for river and distilled water respectively, pointing out a proportion of cells sensitive to low-temperature conditions. Thereafter, progressively and significant stronger losses in culturability occurred, with values below detection level (10^1^ CFU/mL) by 40 ± 7 and 20 ± 3 days, depending on the water sample, in river and distilled water, respectively (Figure 1). These drops in culturable counts with high numbers of cells still viable indicated a majority of the populations becoming VBNC. The strain IVIA-1602.1 displayed similar trends in the microcosms of the other environmental water samples at 4 °C, only with differences in non-culturability between environmental and distilled water (*p* < 0.05). The non-selective medium YPGA and the semiselective SMSA medium yielded similar results for each of the water samples (*p* > 0.05).

At temperate (14 °C) and warm (24 °C) temperatures, trends in total, viable and culturable populations were similar (*p* > 0.05) (Figure 1 and Figure S1), and so only those at 24 °C have been plotted in Figure 1. At both temperatures, total populations of the strain IVIA-1602.1 remained above 10^7^ cells/mL in environmental water and around this value in distilled water, and viability was slightly lower in both types of water for the 40-day experiments. During the period, culturability remained roughly at 10^7^ CFU/mL in environmental water while in distilled water culturable cells stabilized below this value (Figure 1). Assays with the other water samples yielded analogous results, also on both media (*p* > 0.05).

**Figure 2:**
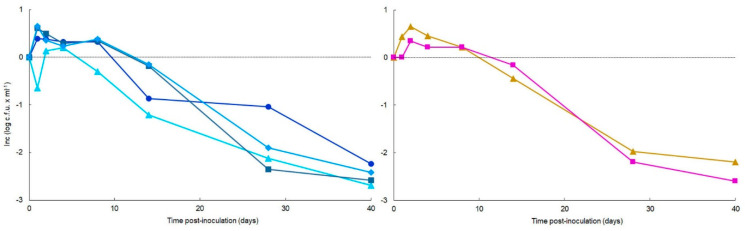
Similarity in trends of culturable cell counts from *Ralstonia solanacearum* strain IVIA-1602.1-inoculated environmental water microcosms throughout 40-day periods at 4 °C and 24 °C. Inc stands for Increments, which were calculated with the differences between mean values of culturable data at both temperatures with respect to the values at time zero (Inc zero). (**Left**): comparison of culturable data on SMSA medium among environmental water (EW)—1 (♦), EW—2 (●), EW—3 (▲), and EW—4 (■). (**Right**): comparison between culturable data on SMSA (■), and YPGA (▲) media for the four environmental water samples.

Similarity in trends of culturable data from the microcosms of the four water samples inoculated with the *R. solanacearum* strains could be observed by the statistical analyses. For comparative purposes, increments of culturable data at 4 °C and 24 °C were jointly calculated with respect to the initial value and plotted with time to assess the effect of water sample (Figure 2, left) and the effect of media (Figure 2, right).

Population dynamics of total, viable, and culturable cells of the strain IPO-1609 of *R. solanacearum* were similar to those of the strain IVIA-1602.1 (*p* > 0.05) in triplicate microcosms from environmental water samples (Figure S2).

### 3.2. R. solanacearum Changes Their Shape in Environmental Water with Increased Temperatures

**Figure 3:**
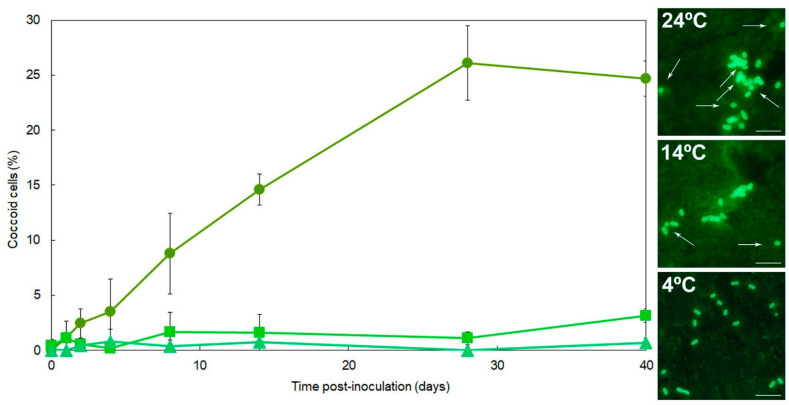
Proportions of coccoid cells appearing in *Ralstonia solanacearum* strain IVIA-1602.1 populations starved in the environmental water microcosms during 40-day periods at 24 °C, 14 °C, and 4 °C. (**Left**) Symbols for each temperature: 24 °C (●), 14 °C (■), and 4 °C (▲). Data from one representative environmental water sample have been plotted. Points are mean ± standard deviation of triplicate microcosms. (**Right**) Representative fluorescence microscopy images of proportions of coccoid cells (white arrows) of the bacterium at each temperature. Scale bars: 5 µm.

Cells of the strain IVIA-1602.1 were examined in triplicate environmental water sample microcosms at 4 °C, 14 °C, and 24 °C. Data are plotted in Figure 3.

At 4 °C and throughout the 40-day experiments, bacterial cells showed the typical *R. solanacearum* bacillar morphology and coccoid cells were seldom detected, with a frequency <1% depending on the water sample (Figure 3).

At 14 °C, a great majority of *R. solanacearum* cells kept bacillar shape. Coccoids were observed in a low proportion, with constant percentages around a value between 1–3% throughout the first 28 days, and then a slight increase up to values ranging 2–6% by 40 dpi, depending on the water sample (Figure 3).

At 24 °C *R. solanacearum* bacilli remained a majority but, coccoid cells were more frequent: depending on the water sample, percentages by the first week were around 8–10%, progressively increasing to 13–16% by the second week, and then up to 22–32% by 28 dpi which stabilized to the end of the 40 days (Figure 3).

Among the low, temperate, and warm temperatures, the average percentage of coccoids significantly increased with temperature (*p* < 0.05). Cell shape of the strain IPO-1609 showed the same trends in one-off trials at each of the three temperatures.

### 3.3. Starved and/or Cold-Induced VBNC R. solanacearum in Environmental Water Keeps Virulent in Planta

**Figure 4:**
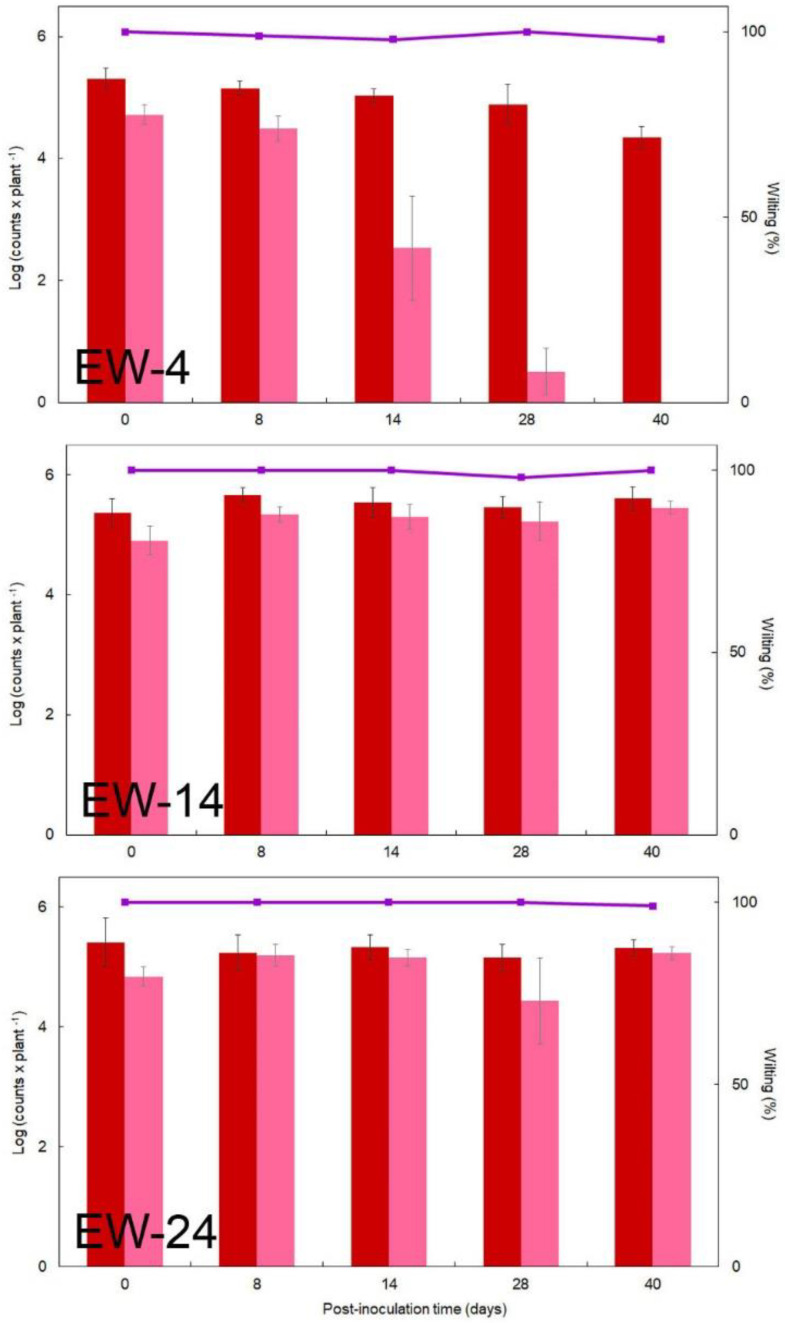
Pathogenicity of *Ralstonia solanacearum* strain IVIA-1602.1 previously starved in environmental water microcosms during 40 days, in tomato plants cv. ‘Roma’. Viable (**red bars**) and culturable (**pink bars**) cells per plant, and percentage of wilted plants (■). Only assays performed in at least weekly intervals from one representative environmental water (EW) at 4 °C (EW−4), 14 °C (EW−14) and 24 °C (EW−24) have been plotted. Points are mean ± standard deviation (SD) of triplicate microcosms. Absolute value for 100% wilting refers to 24 plants (6 × 4 sets). At the three temperatures, SD of wilting values for most of the points was zero, and ± 2.0% in some cases. Control plants inoculated with freshly grown *R. solanacearum* strain IVIA-1602.1 developed 100% wilting, while those inoculated with PBS were negative.

During the 40-day periods of incubation at 4 °C, 14 °C, and 24 °C in the water microcosms under starvation conditions, aliquots were taken at different times to be inoculated in planta. *R. solanacearum* cells of strain IVIA-1602.1 incubated at 4 °C and inoculated in tomato stems induced disease in 98–100% of the plants (Figure 4). Similar wilting percentages were obtained with cells from microcosms at 14 °C and at 24 °C (Figure 4) and were comparable to those of the strain IPO-1609 in one-off trials. At the three temperatures and depending on the water sample, viable *R. solanacearum* cells inoculated per plant were about 10^5^ throughout the 40-day sampling periods, and only from approximately 28 dpi at 4 °C there was a slight decline to values around 10^4^ viable cells per plant (Figure 4). At 4 °C, culturable cells inoculated per plant ranged from 10^5^ to 10^4^ in the initial dpi depending on the water sample. Then, they were decreasing until 10^4^–10^3^ CFU per plant by the first week, and progressively to <10 CFU per plant by 28 dpi and to undetectable levels by 40 dpi (Figure 4). At 14 °C and 24 °C, culturable cells were 10^5^–10^4^ per plant throughout the sampling periods (Figure 4). Plants started to show symptoms within 8–11 dpi and completely wilted within four weeks. The pathogens were re-isolated on SMSA agar from the diseased plants and PCR-identified. Positive control plants yielded 100% wilting. Negative control plants did not show any symptoms.

### 3.4. R. solanacearum Resuscitates from the Cold-Induced VBNC State in Environmental Water and Is Fully Pathogenic in the Host

**Figure 5:**
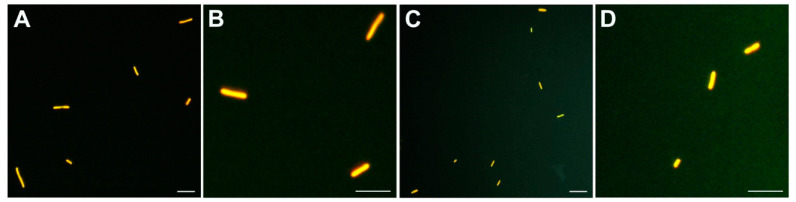
Viability of *Ralstonia solanacearum* strain IVIA-1602.1 populations at 40 days post-inoculation in the environmental water microcosms. (**A**,**B**) starved cells at 24 °C, and (**C**,**D**) starved and cold-induced VBNC cells at 4 °C. Viability was similar between starved (**A**,**B**) and VBNC (**C**,**D**) *R. solanacearum* cells, although in (**C**,**D**) cells were no culturable on solid media. For VBNC cells, this time was considered time zero of VBNC induction of the *R. solanacearum* populations in the resuscitation assays. Viability was measured by the DVC method and subsequent staining with acridine orange [41,42], and estimated in 10^6^ VBNC cells/mL. Scale bars: 5 µm.

Assays carried out to assess the resuscitation capability of the VBNC *R. solanacearum* cells of strain IVIA-1602.1 yielded similar results in triplicate microcosms from environmental water samples. Data are summarized in Table 1, in the three different conditions. The viability of the VBNC cells when the microcosms were containing approximately 10^6^ viable cells/mL and <10 culturable cells (CFU)/mL is illustrated in Figure 5.

#### 3.4.1. By Enrichment in WB Broth

From cold-induced VBNC cells of *R. solanacearum* strain IVIA-1602.1, and after the temperature upshift with shaking and nutrients, monitoring was of: (i) turbidity by *R. solanacearum* growth in the direct aliquots and their serial ten-fold dilutions, (ii) culturability on YPGA, and (iii) pathogenicity in the host. These were observed in all the direct aliquots and their serial ten-fold dilutions up to 10^−6^, corresponding to 1 VBNC cell/mL, at time 0 of the VBNC induction of the *R. solanacearum* populations (Table 1). Thereafter, the resuscitation capability of these VBNC cells in the microcosms was decreasing with time until reaching about two orders of magnitude by one month from the VBNC induction. Time for resuscitation (estimated as time for observation of turbidity by *R. solanacearum* growth) was 24 h for direct aliquots, 36 h for dilutions 10^−1^, 10^−2^, and 10^−3^, 48 h for dilutions 10^−4^ and 10^−5^, and four days for dilutions 10^−6^. These rates of growth were maintained throughout the experimental period. Culturability of the cells in the turbid dilutions was positive in all cases, and colonies were PCR-identified as *R. solanacearum*. However, cells from aliquots in WB broth maintained at 4 °C without shaking remained non-culturable. Pathogenicity assays were positive in all plants, either when inoculated directly from the turbid dilutions or from the colonies on the plates. The pathogen was re-isolated from the wilted plants and PCR-identified. Sensitivity of the detection of resuscitated *R. solanacearum* cells from the VBNC state was 1 VBNC cell/mL.

#### 3.4.2. By Temperature Upshift in Environmental Water

From cold-induced VBNC cells of *R. solanacearum* strain IVIA-1602.1 and after the temperature upshift, monitoring was performed regarding: (i) culturability on YPGA and (ii) pathogenicity in the host. These were observed in cells from the direct aliquots and their serial ten-fold dilutions up to 10^−4^, corresponding to 10^2^ VBNC cells/mL, and up to 10^−3^, corresponding to 10^3^ VBNC cells/mL, respectively, at time 0 of the VBNC induction (Table 1). Then, the resuscitation capability of the cells in the microcosms was decreasing with time until about three orders of magnitude by one month from the induction (Table 1). Time for resuscitation (estimated as time for observation of culturability after plating) was 48 h from the temperature upshift. This was observed after plating 100-µL volumes from the same direct aliquots incubated during 24 h, 48 h and 72 h from the temperature upshift, and then on plates for 3 days at 29 °C. Sampling at 24 h yielded no growth on the plates, sampling at 48 h turned out in countable colonies, and continuous bacterial growth was observed after sampling at 72 h from the temperature upshift, reaching in all cases 10^6^ CFU/mL. Colonies were PCR-identified as *R. solanacearum*. Pathogenicity assays to test cells from the colonies were positive in all plants. Pathogenicity assays to test cells from the direct aliquots and their serial ten-fold dilutions were positive in at least one of the two inoculated plants per microcosm. The pathogen was re-isolated and PCR-identified from the wilted plants. Sensitivity of the detection of resuscitated *R. solanacearum* cells from the VBNC state was 10^2^ VBNC cells/mL.

#### 3.4.3. *In Planta*

From cold-induced VBNC cells of *R. solanacearum* strain IVIA-1602.1 and after the temperature upshift in the host, pathogenicity was monitored. This was observed in cells from the direct aliquots and their serial ten-fold dilutions up to 10^−3^, corresponding to 10^3^ VBNC cells/mL (10 VBNC cells/plant), at time 0 of the VBNC induction, and then the resuscitation capability was decreasing until about one order of magnitude by one month from the induction (Table 1). The pathogen was re-isolated and PCR-identified from the wilted plants. Sensitivity of the detection of resuscitated *R. solanacearum* cells from the VBNC state was 10^3^ VBNC cells/mL.

## 4. Discussion

To anticipate the spread of the disease, current prevention and control strategies against bacterial wilt should take into account knowledge on the potential behavior of *R. solanacearum* in response to global warming. In this work, adaptations by strains of the pathogen from different climatic regions were observed under exposure to environmental temperatures in oligotrophic freshwater, which allowed survival without losing wilting capacity.

**Figure 6:**
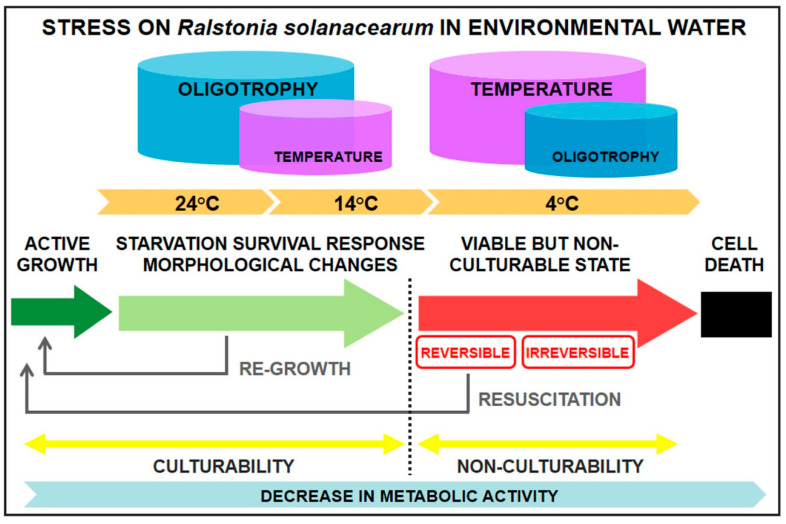
Influence of temperature and oligotrophy on *Ralstonia solanacearum* stress induction in environmental water and stress responses by the bacterium. Adapted to *R. solanacearum* from a proposed model by [67]. See text for details.

With respect to low temperatures, this is the first report of viable *R. solanacearum* populations induced to the VBNC state in environmental water, in conditions more approaching those of natural settings. Previous work described either: (i) a loss in *R. solanacearum* culturability under low temperature in natural or distilled, ultrapure water, but without determining the viability of the bacterial populations, thus without confirming the presence of VBNC cells [26,27,32,46]; or (ii) the VBNC induction in distilled, ultrapure, non-environmental water [28,29]. The fact that other cold-adapted water bacteria are not likely to be cold-induced VBNC [47], contrarily to what was observed in this work with *R. solanacearum* from different climates, suggests that this pathogen is not naturally cold-adapted, even when introduced to cold habitats. Thus, this work demonstrated that, in *R. solanacearum*, low temperature plays a major role than starvation in inducing the VBNC state (Figure 6), contrarily to what has been reported for other bacteria [47]. However, starvation-induced stress proteins could have protected *R. solanacearum* from temperature damage, since it was less vulnerable to cyclic cold stress in pure water than in host tissue [27]. Moreover, the VBNC *R. solanacearum* cold-induction period occurred more slowly in environmental water, pointing to an effect of water nutrient contents, namely trace organic matter and some dissolved salts available for the cells but absent in distilled water, and so nutrient concentrations not supporting *R. solanacearum* growth would act as an additional stress contributing to the cold-induced VBNC state. In the field, latent VBNC cells maintain structure, biology, and significant gene expression, and global climate change might be resuscitating them when low temperature is the inducing factor, leading to increased outbreaks [48]. Similar to *R. solanacearum*, a lower mineral salt concentration markedly shortened the VBNC *Vibrio parahaemolyticus* induction period [49]. Bacterial species, such as *V. vulnificus* and *Aeromonas hydrophila*, also behaved similarly to *R. solanacearum* under low-temperature and nutrient-limiting conditions [50,51], whilst others, such as *Campylobacter jejuni* and *Erwinia amylovora*, displayed different responses [52,53,54,55].

At temperate and warm temperatures in environmental water, *R. solanacearum* populations displayed starvation-survival responses as described at 24 °C [6] and similar in terms of population levels. Lack of unculturability was in agreement with previous work reporting the isolation and persistence of the pathogen in environmental water at temperatures allowing *R. solanacearum* multiplication [15,16,17,26]. The presence of organic matter and salts in environmental water contributed to stimulate *R. solanacearum* survival, similarly to *Aerobacter aerogenes* [56], *E. amylovora* [53,54], and *Leuconostoc mesenteroides* [57], where trace minerals facilitated culturability, since mineral salts can affect not only cell growth, but also cell survival during nutrient limitation conditions [31].

Morphological changes are a visible indicator of adaptation to the environment [31,54,58]. Starved *R. solanacearum* cells transformed from the typical bacilli into coccoids, since shape rounding off and size reduction allow nutrients to be sequestered more efficiently [31]. This was observed in different proportions according to temperature. Although cells entering the VBNC state often exhibit dwarfing [37,59], *R. solanacearum* coccoids were seldom observed during this process, probably because low temperature rapidly causes decrease in *R. solanacearum* metabolism and uptake of water nutrients, with constitutive expression of genes associated with survival and stress response for a stable maintenance of their transcript level [60]. Likewise, copper-induced VBNC *R. solanacearum* cells were unchanged in size [61]. Therefore, at both starvation- and survival-inducing temperatures, the transition to coccoids would be mostly influenced by nutrient limitation and to a lesser extent by low temperature, as reported elsewhere [62]. At these two temperatures, the proportions of coccoids differed, with significantly higher numbers at warm temperature, probably to improve the speed for exchange of material with the surrounding environment to hold a faster energy-consuming metabolism, which becomes a requirement at elevated temperatures [58,63]. Thus, in natural nutrient-deprived environments, the stress of oligotrophy would be less intense for the pathogen at temperatures around 14 °C than at values nearer to the optimum as 24 °C, and so temperature would be modulating this adaptation to oligotrophy (Figure 6), acting on cell metabolism rate and nutrient requirement frequency. Moreover, in the presence of indigenous microbiota, *R. solanacearum* survived longer at 14 °C than at 24 °C in oligotrophic environmental water [35], and the culturability of *R. solanacearum* strain IPO-1609 was favored at 12 °C and 20 °C rather than at 28 °C in agricultural water in both the presence and absence of other aquatic microorganisms [26]. Similar to *R. solanacearum*, a number of bacterial species decreased their sizes with increasing environmental temperatures [51,52,63]. Notwithstanding, this cannot be considered a general bacterial behavior [47,50,58,63].

Although *R. solanacearum* has frequently been described as cold tolerant [14,28], the strains introduced to either cold or warm areas were apparently better temperate-adapted than cold-adapted as considered [12], and similarly to [28], where data indicated that *R. solanacearum* had no special adaptation to survive cold temperatures in water under controlled conditions. Likewise, the cold-water-adapted *Vibrio tasmaniensis* did not enter the VBNC state at 4 °C while the warm-water-adapted *V. shiloi* did [47].

*R. solanacearum* resuscitation from the cold-induced VBNC state was observed after stress removal by placement of the VBNC cells in three different favorable conditions, including the host plant, and all of them implying, at least, an upshift in temperature. In enrichment conditions, culturability on solid medium and pathogenicity of the resuscitated *R. solanacearum* cells from the dilutions were both confirmed. Restoration of culturability was more dependent on the temperature upshift and shaking than the presence of nutrients, since VBNC cells in enrichment liquid medium at 4 °C were not able to form colonies, and so they maintained their VBNC status. In environmental water and after the temperature upshift, culturability and pathogenicity of the resuscitated cells were similarly confirmed, the only resuscitation-inducing factor here being the temperature upshift, which has not been reported for *R. solanacearum* up until now. That would explain the seasonal variation of *R. solanacearum* populations in environmental water [16,17]. If temperature is so critical, increases in water temperature corresponding to rising global surface temperatures will likely lead to a wider geographic distribution of *R. solanacearum* and a higher incidence of infections in planta resulting from resuscitated cells of the pathogen, as it is being observed in *Vibrio* species [48]. Likewise, a simple reversal of temperature was sufficient to allow the resuscitation of other bacterial species [37,38,59]. In contrast, it was not effective to resuscitate the close *R. pseudosolanacearum* (former *R. solanacearum* phylotype I) in soil and water [60,64], since the addition of hydrogen peroxide-degrading compounds, such as catalase or sodium pyruvate, was necessary. The number of bacterial cells resuscitated by temperature upshift in environmental water was equal to the initial inoculum, similarly to [37,44,60]. Resuscitation in planta of the VBNC *R. solanacearum* cells was evidenced by the occurrence of wilting symptoms, and progressively declined over time, accordingly to [29]. Virulence in tomato plants was also observed in revived cells of *R. pseudosolanacearum* after exiting a cold-induced VBNC state in pure water [64]. In all the three different resuscitation conditions, the resuscitated *R. solanacearum* cells displayed similar phenotypes to the original culturable cells, including virulence in tomato plants, as described [60]. Moreover, also in the three different conditions, a decrease in the proportion of VBNC cells capable of resuscitation occurred over time, this process being dependent on the age of the VBNC cells, as stated [38]. In that respect, several authors agree to consider the existence of gradual stages within the VBNC state, namely a reversible non-culturable stage where cells can be resuscitated and an irreversible non-culturable stage, where cells cannot be resuscitated, although they keep respiratory activity [28,60,64] (Figure 6). Among these conditions, in this work, the enrichment was the most effective for *R. solanacearum* resuscitation and the most sensitive for their detection. This is probably because it combines a temperature upshift with nutrients and shaking, which supplies with oxygen and disperses oxidative compounds (peroxides, other free radicals) accumulated extra- or intracellularly either produced in the cells in response to low temperature stress or commonly present in rich culture media [37,65]. Reversal of adverse VBNC-state-inducing factors can be efficiently applied to the detection of *R. solanacearum* resuscitated cells.

On the basis of all these results, starved and/or cold-stressed and/or cold-induced VBNC *R. solanacearum* cells could be present in environmental water, being a threat to secure crop production as they are not easily detected [61], can survive cold temperature fluctuations [27], and can revert to a fully pathogenic state just by a temperature upshift, which can be favored within the frame of global climate change conditions. All of these *R. solanacearum* survival forms maintained their capacity for in planta multiplication and colonization, causing disease symptoms in the host, as observed elsewhere for similar time periods [6,16,29]. Not only can global warming contribute to the pathogen spread and virulence, but crops resistant to *R. solanacearum* at moderate temperatures can also become more susceptible at high ambient temperatures [14,24], increasing the probability of infections. The bacterial wilt disease is most severe on plants at temperature values ranging from 25 °C to 35 °C [14,28,66].

On the other hand, taking into account the temperature interval of 4–10 °C applied by EU legislation [43] to transport suspected water samples, temperatures above 4 °C up to around 10 °C would be more advisable than 4 °C, since cultivation-based methods are required to confirm pathogen detection. For the inspection of environmental samples, it should be determined whether to also test for these VBNC cells [64] to improve the sensitivity of the detection. This is a relevant point since the early detection of *R. solanacearum* in irrigation water and its eradication would contribute to improve any integrated management program of the bacterial wilt disease [61].

Overall, *R. solanacearum* strains from either cold or warm origin were able to adapt to a combined effect of temperature and oligotrophy. At low temperature, the delay in the induction of the VBNC state in environmental water suggested a protective effect of water nutrient contents on bacterial cells and pointed out the relevance of performing survival studies in conditions better approaching those in the environment. At temperate and warm temperatures, adaptations to oligotrophy were starvation–survival responses and morphological changes influenced by temperature. It appeared that, when temperature was the main stress (cold conditions), nutrient deprivation acted as an additional stress, contributing to accelerate the effect of temperature, and conversely, when oligotrophy was the main stress (temperate and warm conditions), temperature increased the effect of oligotrophy (Figure 6). In all conditions, *R. solanacearum* cells remained pathogenic and capable of resuscitation by a simple reversal of temperature.

Under circumstances of global warming, understanding *R. solanacearum* adaptations to environmental abiotic stresses can help to design strategies to prevent and control their spread and dissemination in waterways and other natural settings. This is particularly important in the case of *R. solanacearum*-contaminated water, since it cannot be used for irrigation, contributing to the global problem of the increased water scarcity in the environment due to climate change, which has serious implications, among others, for food production and health.

## Figures and Tables

**Table 1:** Resuscitation of *Ralstonia solanacearum* strain IVIA-1602.1 previously induced to the VBNC state in environmental water microcosms at 4 °C.

Time in the VBNC State (Weeks)	Culturable Cells (CFU/mL)		Resuscitation Assays								
			*In Vitro*–In Enrichment Conditions								
			Direct	−1	−2	−3	−4	−5	−6	−7	−8
0	3	Turbidity	3/3	3/3	3/3	3/3	3/3	3/3	3/3	0/3	0/3
		Culturability	3/3	3/3	3/3	3/3	3/3	3/3	3/3	0/3	0/3
		Pathogenicity	3/3	3/3	3/3	3/3	3/3	3/3	3/3	0/3	0/3
1	0	Turbidity	3/3	3/3	3/3	3/3	3/3	3/3	0/3	0/3	
		Culturability	3/3	3/3	3/3	3/3	3/3	3/3	0/3	0/3	
		Pathogenicity	3/3	3/3	3/3	3/3	3/3	3/3	0/3	0/3	
2	0	Turbidity	3/3	3/3	3/3	3/3	3/3	3/3	0/3		
		Culturability	3/3	3/3	3/3	3/3	3/3	3/3	0/3		
		Pathogenicity	3/3	3/3	3/3	3/3	3/3	3/3	0/3		
3	0	Turbidity	3/3	3/3	3/3	3/3	3/3	2/3	0/3		
		Culturability	3/3	3/3	3/3	3/3	3/3	2/3	0/3		
		Pathogenicity	3/3	3/3	3/3	3/3	3/3	2/3	0/3		
4	0	Turbidity	3/3	3/3	3/3	3/3	3/3	0/3	0/3		
		Culturability	3/3	3/3	3/3	3/3	3/3	0/3	0/3		
		Pathogenicity	3/3	3/3	3/3	3/3	3/3	0/3	0/3		
			***In Vitro*–In Environmental Water**								
			**Direct**	**−1**	**−2**	**−3**	**−4**	**−5**	**−6**	**−7**	**−8**
0	3	Culturability	3/3	3/3	3/3	3/3	3/3	0/3	0/3	0/3	0/3
		Pathogenicity	3/3	3/3	3/3	3/3	3/3	0/3	0/3	0/3	0/3
2	0	Culturability	3/3	3/3	3/3	1/3	0/3	0/3			
		Pathogenicity	3/3	3/3	3/3	1/3	0/3	0/3			
4	0	Culturability	3/3	2/3	0/3	0/3	0/3				
		Pathogenicity	3/3	2/3	0/3	0/3	0/3				
			***In Planta***								
			**Direct**	**−1**	**−2**	**−3**	**−4**	**−5**			
0	3	Pathogenicity	3/3	3/3	3/3	3/3	0/3	0/3			
4	0	Pathogenicity	3/3	3/3	2/3	0/3	0/3	0/3			

Data from one representative environmental water at 4 °C (EW−4) are summarized. Direct stands for direct aliquots, and the negative numbers stand for the serial ten-fold dilutions. Pathogenicity assays were considered positive when at least one of the two tomato plants cv. ‘Roma’ inoculated per microcosm showed bacterial wilt symptoms. *R. solanacearum* was re-isolated from wilted plants and PCR-identified. Control plants inoculated with *R. solanacearum* strain IVIA-1602.1 at each sampling time yielded 100% wilting in stem inoculation. Plants inoculated with PBS were negative. Data represent results from triplicate microcosms.

## Data Availability

Not applicable.

## Supplementary Materials

The following supporting information can be downloaded at: https://www.mdpi.com/article/10.3390/microorganisms10122503/s1, Figure S1: Effect of temperature under nutrient-limiting conditions on survival of *Ralstonia solanacearum* strain IVIA-1602.1 during 40-day periods in environmental water at 14 °C. Total (■), viable (●), and culturable cells on SMSA (▲) and YPGA (♦) media. Points are mean ± standard deviation of triplicate microcosms; Figure S2: Effect of low temperature under nutrient-limiting conditions on survival of *Ralstonia solanacearum* strain IPO-1609 during 40-day periods in water. Microcosms of environmental water at: 4 °C (top), 14 °C (middle), and 24 °C (bottom). Total (■), viable (●), and culturable cells on SMSA (▲) and YPGA (♦) media. Points are mean ± standard deviation of triplicate microcosms.
