# Supplementary material for: Ralstonia solanacearum Facing Spread-Determining Climatic Temperatures, Sustained Starvation, and Naturally Induced Resuscitation of Viable but Non-Culturable Cells in Environmental Water
Source: Microorganisms. 2022 Dec 16;10(12):2503. doi: 10.3390/microorganisms10122503 (PMC9784099; doi:10.3390/microorganisms10122503)
Supplement: Supplementary file 1 [file microorganisms-10-02503-s001.zip › Figure S1.pdf]

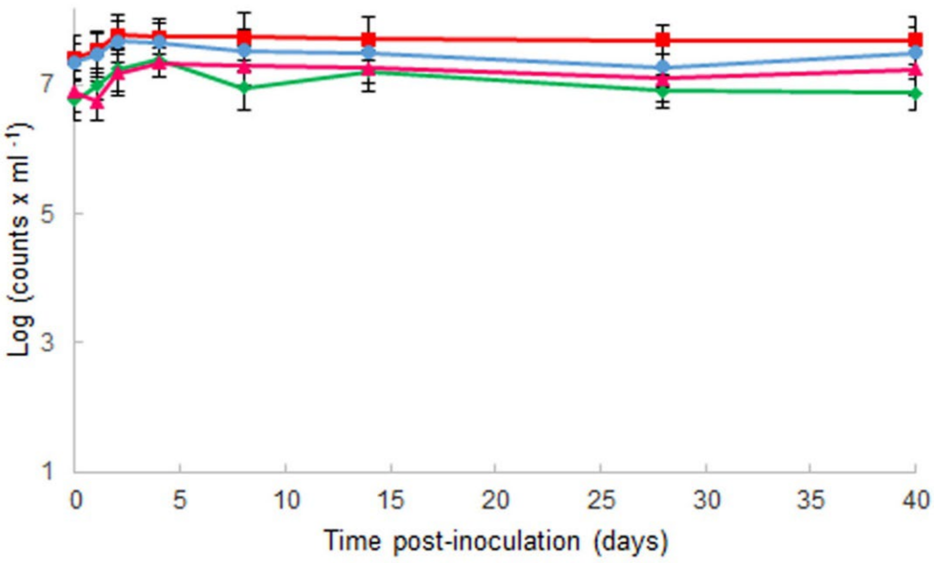

Figure S1. Effect of temperature under nutrient-limiting conditions on survival of *Ralstonia solanacearum* strain IVIA-1602.1 during 40-day periods in environmental water at 14°C. Total (■), viable (●), and culturable cells on SMSA (▲) and YPGA (♦) media. Points are mean ± standard deviation of triplicate microcosms.
