# Supplementary material for: Ralstonia solanacearum Facing Spread-Determining Climatic Temperatures, Sustained Starvation, and Naturally Induced Resuscitation of Viable but Non-Culturable Cells in Environmental Water
Source: Microorganisms. 2022 Dec 16;10(12):2503. doi: 10.3390/microorganisms10122503 (PMC9784099; doi:10.3390/microorganisms10122503)
Supplement: Supplementary file 1 [file microorganisms-10-02503-s001.zip › Figure S2.pdf]

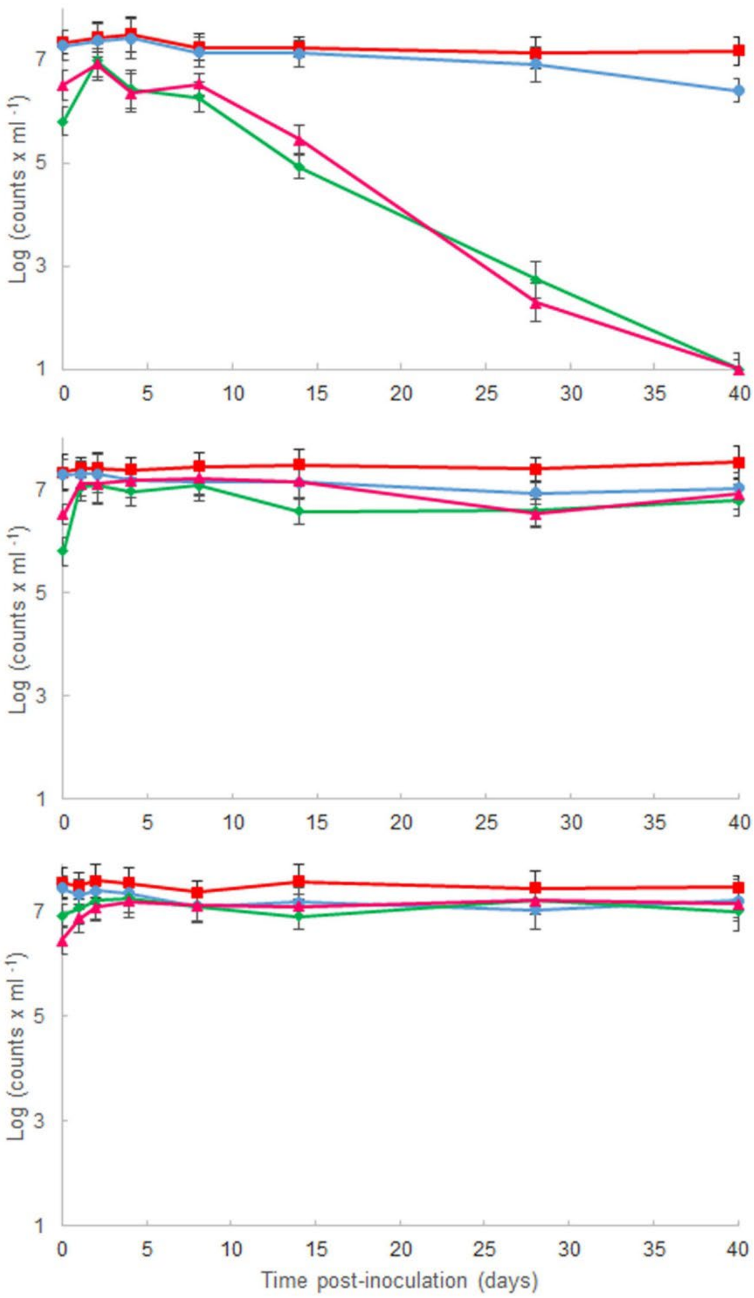

Figure S2. Effect of low temperature under nutrient-limiting conditions on survival of *Ralstonia solanacearum* strain IPO-1609 during 40-day periods in water. Microcosms of environmental water at: 4°C (top), 14°C (middle), and 24°C (bottom). Total (■), viable (●), and culturable cells on SMSA (▲) and YPGA (♦) media. Points are mean ± standard deviation of triplicate microcosms.
